# Personal, social, and environmental factors associated with lifejacket wear in adults and children: A systematic literature review

**DOI:** 10.1371/journal.pone.0196421

**Published:** 2018-05-02

**Authors:** Amy E. Peden, Daniel Demant, Martin S. Hagger, Kyra Hamilton

**Affiliations:** 1 Royal Life Saving Society - Australia, Sydney, Australia; 2 College of Public Health, Medical and Veterinary Sciences, James Cook University, Townsville, Australia; 3 Faculty of Health, University of Technology Sydney, Ultimo, New South Wales, Australia; 4 Institute of Health and Biomedical Innovation, Queensland University of Technology, Brisbane, Australia; 5 School of Applied Psychology and Menzies Mental Health Institute Queensland, Griffith University, Brisbane, Australia; 6 School of Psychology and Speech Pathology and Health Psychology and Behavioural Medicine Research Group, Curtin University, Perth, Australia; 7 Faculty of Sport and Health Sciences, University of Jyväskylä, Jyväskylä, Finland; Edith Cowan University, AUSTRALIA

## Abstract

**Objective:**

Drowning claims 7% of the global burden of injury-related deaths. Lifejackets are routinely recommended as a drowning prevention strategy; however, a review of related factors regarding lifejacket wear has not previously been investigated.

**Methods:**

This systematic review examined literature published from inception to December 2016 in English and German languages. The personal, social, and environmental factors associated with lifejacket wear among adults and children were investigated, a quantitative evaluation of the results undertaken, and gaps in the literature identified.

**Results:**

Twenty studies, with sample sizes of studies ranging between 20 and 482,331, were identified. Fifty-five percent were cross-sectional studies. All studies were scored IV or V on the Australian National Health and Medical Research Council (NHMRC) grading system indicating mostly descriptive and cross-sectional levels of evidence. Factors associated with increased wear included age (mostly children), gender (mostly female), boat type (non-motorised), boat size (small boats), role modelling (children influenced by adult lifejacket wear), and activity (water-skiing, fishing). Factors not associated or inconsistent with lifejacket wear included education, household income, ethnicity, boating ability, confidence in lifejackets, waterway type, and weather and water conditions. Factors associated with reduced lifejacket wear included adults, males, discomfort, cost and accessibility, consumption of alcohol, and swimming ability. Three studies evaluated the impact of interventions.

**Conclusion:**

This review identified factors associated with both increased and decreased lifejacket wear. Future research should address the motivational factors associated with individuals’ decisions to wear or not wear lifejackets. This, combined with further research on the evaluation of interventions designed to increase lifejacket wear, will enhance the evidence base to support future drowning prevention interventions.

## Introduction

Drowning is a risk associated with aquatic activity [1, 2] and is estimated to claim the lives of 372,000 people every year [3], accounting for 7% of the global burden of injury-related death. While 97% of unintentional drownings occur in low and middle-income countries (LMIC) as a result of the activities of daily life [4]; in high income countries, fatal drowning is often a result of recreational activities such as swimming, fishing, and watercraft use [5]. In Australia, 20% of all unintentional fatal drownings are due to watercraft incidents; the second leading cause of drowning in Australia after swimming [5].

Advocating best practice in legislation, enforcement, and promotion of lifejacket wear is identified within the Australian Water Safety Strategy 2016–2020 as a key objective for reducing boating and watercraft-related drowning deaths [6]. The enforcement of legislation regarding lifejacket provision and wear is believed to be an effective prevention measure [7] for deaths as a result of boating, shipping, and ferry-related incidents [3]. Systematic reviews have identified lifejackets as a drowning prevention strategy for rivers [8], adults [9], and older people [10]. Lifejackets can also be used as a drowning prevention strategy for young children, weak swimmers, and those who fish from rocks or a boat [7].

Not wearing a lifejacket may increase drowning risk [8]; studies find generally low levels of lifejacket wear among drowning victims [11, 12] and among some cultural groups [13]. An ongoing drowning prevention challenge has been increasing lifejacket wear rates. Regulation and associated enforcement are common strategies used to increase the use of lifejackets [7, 14, 15]; however, other factors may also contribute to increased wear rates. To develop effective interventions for lifejacket wear, influences on, and determinants of, lifejacket wear rates need to be well-understood. Data from cross-sectional studies of association can help identify potential predictors of lifejacket wear that can be targeted for behaviour change in interventions. Baranowski et al. [16] argue that interventions which target strong and consistent modifiable correlates of behaviour should be more effective in changing behaviour. To date, there has been no synthesis of the literature on the associations toward lifejacket wear. The aim of this systematic review was to comprehensively evaluate published studies on personal, social, and environmental factors associated with lifejacket wear. The review makes a quantitative evaluation of the results, identifies gaps in the literature and directions for future research.

## Materials and method

**Fig 1:**
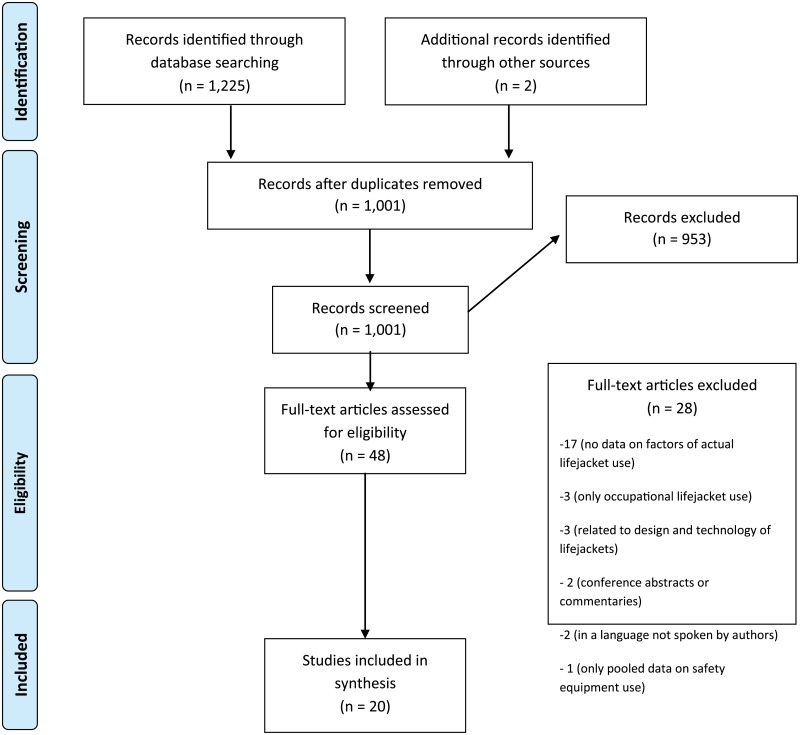
Flow diagram.

We followed published reporting guidelines [17, 18] adapted for narrative reviews. The PRIMSA checklist is provided (Fig 1) (S1 Table).

### Search strategies and databases

Databases SCOPUS (contains Medline), PsychINFO, and Web of Science were searched in November and December 2016 for relevant sources (from inception). The search terms used were personal flotation device OR personal floatation device OR life vest OR lifevest OR lifejacket OR life jacket OR lifesaver OR life saver OR lifebelt OR life belt OR life preserver OR buoyancy vest OR buoyancy jacket OR buoyancy aid OR buoyancy device. The same search terms were used for all databases. No further restrictions and limits were used for any database. Additionally, bibliographies and reference lists of all included publications were screened for additional references.

### Inclusion and exclusion criteria

All publications had to provide qualitative or quantitative data on difference in lifejacket wear between defined groups or on other potential factors related to lifejacket wear. Studies on occupational lifejacket wear were excluded as were studies on differences between occupational and non-occupational lifejacket wear. Results were limited to the language skills of the authors (English and German). Grey literature was excluded.

### Review, data extraction and quality assessment

All citations were downloaded to citation management software (EndNote X7^™^, Thomson Reuters) and duplicates removed. The second author reviewed titles and abstracts of all references and selected those which met the inclusion criteria for further assessment. A full-text assessment of all remaining articles was conducted independently by two researchers. Where differences in opinion arose, a third researcher reviewed these publications. See Fig 1 for the flow diagram. Subsequently, data from each article was extracted (S2 Table).

The level of evidence of all articles was determined according to the National Health and Medical Research Council (NHMRC) guidelines [19]. The majority of articles received a grade of IV which represents the lowest level of evidence. Qualitative studies were given a grade of V, as they are currently not captured by the NHMRC approach.

### Data analysis

Selected studies varied in sample size, study design, outcomes, and populations. A narrative approach was therefore selected due to the strong heterogeneity in the publications. Data presented in the included studies was extracted and categorised (see S1 Table), and authors reached consensus on the final interpretation of results. Some included publications analysed the effectiveness of regulations and/or interventions. Factors related to regional differences within one country were omitted. Three publications [14, 15, 20] reported on interventions only and presented data specifically for the purpose of analysing the effectiveness of the intervention; differences in lifejacket use by groups was not presented. These studies were analysed separately as ‘interventions’ and not as individual factors. A fourth publication [21] investigating the effectiveness of an intervention, provided data to analyse both potential factors and the effectiveness of the interventions; results were presented as factors and in the separate section on interventions.

### Characteristics of included studies

#### Study designs and populations

This systematic review included 20 publications. Table 1 outlines the main characteristics and outcomes of included studies. Most studies were published within the past 10 years (n = 16) and conducted in North America (n = 16), with two studies being conducted in both Australia and New Zealand. All but two publications were quantitative with most applying a cross-sectional study design (n = 11), followed by population-based (n = 6) and one cohort study employing cross-sectional analysis.

**Table 1:** Characteristics of included studies.

Reference	Country (area within country)	Year(s)*	Type of Study	Population	Sample Size	Factors	Level of Evidence^#^
Bennett et al (1999)	USA (King County, Washington State)	1992–1994	Cross-sectional	Children (1–14 years)	812	Age, Confidence in lifejackets, Comfort of lifejackets, Education, Swimming abilities, Income, Intervention, Ownership of lifejacket, Perception of danger, Role modelling	IV
Bugeja et al (2014)	Australia (Victoria)	1998–2004 & 2005–2010	Population-based	Recreational boating drowning deaths	74	Legislation/Regulation (subanalysis by Age, Boat type, Gender, Waterway type)	IV
Cassell et al (2015)	Australia (Victoria)	2005 & 2007	Cross-sectional (observation)	Occupants of small power recreational vessels	5029	Legislation/Regulation (subanalysis by Age, Boat type, Gender, Type of activity)	IV
Chung et al (2014)	USA (Washington State)	2010	Cross sectional (observation)	Boaters (general)	5157	Legislation/Regulation (analysed by Age, Boat type, Gender, Role modelling, Type of activity, Weather/Water conditions)	IV
Clemens et al (2016)	Canada	2008–2012	Population-based	Drowning fatalities	2392	Age	IV
Croft et al (2015)	New Zealand	1983–2012	Population-based	Adult males (drowning deaths)	2134	Alcohol and other drugs	IV
Dai et al (2013)	USA (Georgia)	2002–2008	Population-based	Children and adolescents (drowning deaths)	220	Race/Ethnicity	IV
Giles et al (2010)	Canada (Tuktoyaktuk, Northwest Territories)	2007	Qualitative (Interviews)	General (mostly First Nations Canadians)	20	Accessibility of lifejackets, Boating abilities, Confidence in lifejackets, Role modelling	V
Jones (1999)	USA (Arkansas)	1994–1997	Population-based	Personal watercraft passengers crash victims	246	Seat position	IV
Mangione et al (2012)	USA	1999–2010	Cross-sectional (observation)	Boaters	482331	Age, Boat type	IV
Mangione et al (2014)	USA (California & Mississippi)	2006–2011	Cross-sectional (observation)	Boaters	109449	Intervention & Legislation/Regulation (with subanalysis by Age, Boat type, Gender, Type of activity)	IV
Moran (2011)	New Zealand	2003	Cross-sectional (survey)	High school students (16–19 years)	2202	Gender	IV
Nathanson et al (2010)	United States	2006	Cross-sectional (survey)	Sailors	1860	Age	IV
Quan et al (1998)	United States (Oregon & Washington)	1995	Cross-sectional (observation)	Boaters	4210	Age, Boat type, Gender, Water/Weather conditions	IV
Quistberg et al (2014a)	United State (western Washington State)	2008	Cross-sectional (survey)	Boaters	675	Accessibility of lifejackets, Age, Alcohol and other drugs, Boat type, Boating abilities, Comfort of lifejackets, Confidence in lifejackets, Education, Gender, Income, Role modelling, Swimming abilities, Type of Activity, Water/Weather conditions, Waterway type,	IV
Quistberg et al (2014b)	USA	2008	Qualitative (Focus groups)	Boaters	16	Alcohol and other drugs, Boating abilities, Comfort of lifejackets, Legislation/Regulation	V
Redwood et al (2009)	USA (Alaska)	2004–2006	Cohort (cross-sectional analysis)	Alaskan and American Indigenous	3828	Age, Gender, Region	IV
Strayer et al (2010)	USA (Alaska)	2000–2006	Population-based	Drowning fatalities	402	Gender, Race/Ethnicity	IV
Treser et al (1997)	USA (King County, Washington State)	1992 & 1994	Cross-sectional	Boaters (small-sized boats)	4088	Age, Gender	IV
Wintemute et al (2013)	USA (Sacramento County, California)	2010	Cross-sectional (observation)	Children (0–13 years)	1739	Age, Gender, Race/Ethnicity	IV

* Time of data collection;

^#^ According to NHMRC (National Health and Medical Research Council)

Populations varied in the sample; 11 studies were specifically concerned with boating populations including sailors and personal watercraft (PWC) users, with two studies focussing on drowning fatalities and crash victims. Four studies focussed on drowning deaths in different populations, three studies focussed on children and adolescents, and two studies focussed predominantly on Indigenous Americans.

Sample sizes also varied, with very low samples in qualitative studies. Sample sizes in studies with cross-sectional designs tended to be higher than among population-based studies. Overall, sample sizes among quantitative studies varied between 74 and 482,331.

#### Outcomes

The Outcomes (factors) were broadly categorised to increase comparability of outcomes (see Table 1). Factors most commonly analysed were demographic in nature; age (n = 10), education and income (n = 1), gender (n = 9), and race/ethnicity (n = 3). Other factors were related to boating including boat type and length (n = 5), boating ability (n = 3), and seat position (n = 1); and lifejackets including comfort (n = 2), accessibility and ownership (n = 3), and confidence (n = 2). Publications also analysed alcohol and other drugs (n = 3), role modelling (n = 2), swimming abilities (n = 1), type of activity (n = 3), waterway types (n = 1), and water or weather conditions (n = 3).

## Results

### Socio-demographic factors

#### Age

In total, ten studies [7, 21–29] provided sufficient data for an analysis by age and/or age group. Most publications reported data on boaters or sailors. Overall, these studies indicated that children were the most likely to wear lifejackets and that young people were, on average, more likely to wear a lifejacket than their older counterparts. Chung et al. [23] found both children and adolescents were significantly more likely to wear lifejackets than adults, whereas differences between age groups among adults were not significant. These results are supported by Mangione et al. [21, 22], and Treser et al. [28]. Furthermore, an observational study on boaters found children were more likely to wear lifejackets than adolescents [26], a finding supported by another observational study in Sacramento County [7].

Similarly, in a study on drowning deaths in Canada [24], victims aged 5 to 14 years were found to be more likely to be wearing a lifejacket than their older counterparts. Adolescents aged 15 to 19 years had the lowest lifejacket wear rate followed by young adults aged 20 to 35 years, however significance levels between groups were not provided. A study of 1,860 sailors [25] also showed that those under the age of 30 years were significantly more likely to use lifejackets than those over the age of 30 years.

Other studies were less consistent, showing an increase in lifejacket use by age among Alaskan and American indigenous adults [27]. Quistberg et al. [29] found differences in lifejacket use by age with those aged 30 to 59 years more likely to wear lifejacket than their younger and older counterparts; these differences were not statistically significant.

#### Education and income

A cross-sectional study on boaters in Western Washington [29] did not show a significant difference in lifejacket use by household income and between those with and without some college education.

#### Gender

A total of nine studies examined the effects of gender on lifejacket use. Four [23, 27, 29, 30] of these studies found females were significantly more likely to wear lifejackets than males. However, two studies in California and Mississippi [21] showed males were consistently more likely to wear lifejackets than females on all intervention and comparison groups at baseline and three post-intervention points, with one slightly higher figure for females in the Mississippi intervention group at three years’ post-intervention; no significance values were provided for differences between males and females. Wintemute et al. [7] did not detect a significant difference in lifejacket wear by gender among children under the age of 14 years, and a study on drowning fatalities in Alaska [31] could not detect a difference in lifejacket wear by gender among victims. Treser et al.’s [28] analysis showed females compared to males were slightly more likely to wear lifejackets; no statistical significance values were provided.

#### Ethnicity

Two of three studies with data on this factor did not identify significant differences in lifejacket wear between ethnicities/races in drowning fatalities [31, 32]. One observational study [7], however, showed children of Asian descent were significantly less likely to wear lifejackets than the comparison group (‘uncertain ethnicity’).

### Boating-related factors

#### Boat type and length

Five studies considered boat type and length as potential factors influencing lifejacket use. Three [22, 23, 26] of these looked specifically at different boat types; individuals on non-motorised boats were significantly more likely to wear lifejackets than those on motorised boats. In Washington State, users of PWC, kayaks, canoes, rowboats, paddleboards/sailboards, and sailboats were significantly more likely to be observed to wear lifejackets than those on motorised boats; differences for users in inflatable boats were higher but not significantly so after adjustments [23].

A large study [22] showed that between 1999 and 2010 people on non-powered boats were observed to wear lifejackets in 20.2% of observations compared to 3.9% on power boats; among those on non-motorised boats, users of kayaks, and sailboards showed the highest figures with 78.6% and 76.7%, respectively, and users on cabin sailboats the lowest with 12.3%. In contrast, the highest figure for users of motorised boats was 8.3% for users of skiff or utility boats. Mangione et al. [22], however, did not provide probability levels for differences between boat types. Similarly, Quan et al. [26] showed that relative to users of motorboats, users of all other types of boats had a higher relative prevalence of wearing lifejackets. Those on kayaks had the highest rate of lifejacket use with 77.6% followed by sailboats with 50%, compared to 19% for motorboats.

Two other studies [21, 29] looked at boat size as a predictor of lifejacket use with inconsistent results. In one study [29], individuals on large boats were more likely to use lifejackets followed by those on medium-sized boats, whereas users of small boats were least likely to use lifejackets. In another study [21], comparing two different interventions to increase lifejacket use, those in small boats were more likely to wear lifejackets than those in medium and large sized boats in California and Mississippi. This continued at post-intervention at all sites except for intervention sites in Mississippi where users of medium sized boats were the most likely to wear lifejackets.

#### Boating abilities

No differences were detected in lifejacket use by self-rated piloting skills, undertaking safety classes, possessing boating education cards, years of boating experience, or frequency of boating [29]. In a qualitative study [33] of Canadians with the majority from First Nations backgrounds, participants perceived the use of lifejackets as optional depending on the boating skills of the person. Lifejacket use was more important for those with low skill levels or less experience in boating. Participants in another qualitative study discussed how less experience may lead to overconfidence and lower levels of lifejacket use [34].

#### Seat position

One study [35] on drowning in Arkansas waterways between 1994 and 1997 showed no difference in the use of lifejackets between operators and riders, with 98% of subjects in each category wearing lifejackets.

### Factors related to lifejackets

#### Comfort of lifejackets

Quistberg et al. [29] found that discomfort was significantly associated with low or no lifejacket use. A further qualitative study supported this argument where lifejackets perceived to be comfortable by participants’, particularly inflatable lifejackets, resulted in increased use [34].

#### Accessibility and ownership of lifejackets

Those with inflatable lifejackets on board were significantly less likely to report no or low use of lifejackets [29]. Furthermore, in two qualitative studies, participants’ perceived lifejackets to be too costly, resulting in low rates of lifejacket ownership and lower rates of lifejacket use [33, 34]. Lifejackets were also perceived to be physically inaccessible in certain geographical areas [33].

#### Confidence in lifejackets

One study [29] found that confidence in lifejackets was not significantly associated with lifejacket use. However, a qualitative study from Canada [33] found that low levels of confidence in the effectiveness of lifejackets may be associated with lifejacket use. In this study, participants’ perceived lifejackets to be ineffective in certain conditions such as cold water and, were therefore, deemed unnecessary.

### Other factors

#### Alcohol and other drugs

Results from a study [36] on adult male drowning deaths in New Zealand showed those not consuming alcohol were more likely to wear lifejackets than those who drank; no probability levels were given and the overall analysis is difficult to interpret. A qualitative study from the US [34] found safety behaviours such as wearing lifejackets were perceived as being of lower importance after individuals’ consumed alcohol. Quistberg et al. [29] also found that alcohol use was significantly associated with no or low lifejacket use among boaters in Washington State.

#### Role modelling

Children and adolescents were more likely to wear lifejackets if an adult in the boat was also wearing a lifejacket, with multivariable incidence risk ratios ranging from 6.2 for children aged 6 to 12 years to 20.0 for adolescents aged 13 to 17 years; the difference being significant only for adolescents aged 13 to 17. Role modelling also affected lifejacket use for adults [29], with adults being less likely to report no or low use of lifejackets if children or adolescents were on board; differences were only significant if children under the age of 10 years were on board. Similarly, a qualitative study [33] described the importance of role modelling in First Nations communities in Canada. However, the behaviour of elders was perceived as having a negative influence on lifejacket use in their community. Younger members of the community perceived elders as role models and refused to wear lifejackets as their elders do not wear them either.

#### Swimming abilities

One study by Quistberg et al. [29] among boaters in Washington State showed no or low use of lifejackets was significantly associated with those perceiving themselves to be intermediate or expert swimmers.

#### Type of activity

Adjusted risk ratios [23] showed no differences in lifejacket use by activity type using fishing (or the intent to fish) as a reference group, with the exception of those undergoing water-skiing activities who were significantly more likely to wear lifejackets. Similarly, Quistberg et al. [29] could not detect a difference in lifejacket wear by activity type. Observations by Mangione et al. [22] showed that those intending to fish were more likely to wear lifejackets than those undertaking other activities at comparison and intervention sites in California and Mississippi at baseline and post-intervention, with one exception for Californian comparison sites one year post intervention. No statistical probability levels were provided.

#### Waterway type

Quistberg et al. [29] did not detect a significant difference in lifejacket use between those in salt water and those in freshwater.

#### Weather and water conditions

A total of three studies [23, 26, 29] looked at weather conditions and lifejacket use with inconsistent results. Quan et al. [26] did not detect a significant difference in lifejacket use between cloudy, partly cloudy, and sunny weather conditions nor did temperature have an influence on lifejacket use. Other studies have found significant differences. Chung et al. [23] found lifejacket use was less likely in partly cloudy or rainy conditions with no significant differences between sunny and cloudy conditions. Quistberg et al. [29] found no or low use of lifejackets was more likely in warmer conditions. Two of these studies [23, 29] analysed the effect of water conditions on lifejacket use; choppy water conditions were found to be negatively associated with lifejacket use in both studies.

### Interventions

Bennett et al. [20] investigated the effects of a drowning prevention campaign aimed at increasing lifejacket use among children and adolescents aged 1 to 14 years in King County, Washington State. The campaign resulted in modest effects on some variables, particularly in reported lifejacket use and ownership among those who could recall the campaign. Another study [14] investigated compulsory lifejacket wearing regulations on drowning deaths among recreational boaters. The Australian State of Victoria introduced mandatory lifejacket use in December 2005. While the analysis did not look specifically at lifejacket use, results showed a significant decline in drownings within the group of interest, potentially as a result of higher lifejacket use. This is consistent with a further observational study [15] looking at the same intervention which showed lifejacket use increased significantly among boaters in small vessels. The results of the aforementioned studies are of particular interest in light of a study by Mangione et al. [21] which compared mandatory regulations (in Mississippi) with educational marketing campaigns (in California). Both approaches showed increased lifejacket use in adults.

## Discussion

This systematic review identified 20 studies detailing the personal, social, and environmental factors associated with lifejacket wear in both adults and children.

### Factors associated with increased lifejacket wear

A range of factors were found to be associated with increased lifejacket wear. These included age (mostly children), gender (mostly female), boat type (non-motorised), boat size (small boats), role modelling (mostly children influenced by adults wearing a lifejacket), and activity (water-skiing, fishing).

Young age was specified as a significant factor for increased lifejacket wear, which may, in part, be due to legislation and regulation mandating lifejacket wear for children [14, 23, 29]. Children generally do not feature in boating-related drowning fatalities [37–40]. Exposure studies are required to determine if children are less exposed to the risk through reduced participation in boating when compared to adults, or if the practice of wearing lifejackets, supported by legislation, is proving to be effective in reducing drowning risk.

Boat type was also found to be associated with increased lifejacket wear, with those using non-motorised boats significantly more likely to wear lifejackets [22, 23, 26]. Qualitative research is required to determine differences in perception of drowning risk and the role of lifejackets between those who use powered and non-powered boats. The impact of boat size was inconsistent [21, 29] with users of large boats and small boats found to have the highest lifejacket wear rate in two studies. Regardless of such findings, small vessels (generally 6 metres and under in length) continue to feature heavily in boating-related incident and fatality data in Australia [41], Canada [42], and the United States [39] and therefore effective strategies for increasing lifejacket wear rates among users of small boats are required.

Role modelling was also identified as being associated with lifejacket wear among children, adolescents, and indigenous communities [23]. Role modelling has been used as an injury prevention strategy in the areas of ski safety [43], bicycle helmet use [44], and seat belt use [45]. Drowning prevention advocates should explore using role models for public awareness, education, and advocacy campaigns to reduce boating-related incidents and evaluate their effectiveness.

Those engaged in water-skiing and fishing were also found to be more likely to wear a lifejacket. Such findings should be validated through the use of exposure studies, and qualitative research should be conducted to understand individuals’ beliefs and attitudes towards the wearing of lifejackets when undertaking these activities. It may be that those engaging in such pursuits have positive attitudes toward the equipment needed to undertake their activities and see lifejackets as essential kit and/or view lifejackets as a facilitator to undertake their chosen activity rather than as an inhibitor.

### Factors associated with decreased lifejacket wear

Factors associated with decreased wear included adults, males, discomfort of lifejackets, cost and accessibility, consumption of alcohol, and swimming ability.

Adult males are overrepresented in drowning fatalities [3, 38] including those as a result of boating and watercraft incidents [37, 40]. Lifejackets are an important part of a drowning prevention strategy for males who boat and use watercraft [29, 37]. Further work is required to fully understand the barriers to lifejacket wear among males, including stratifying this research by age, aquatic location, and activity. The implementation and evaluation of strategies to combat these barriers must also be conducted.

It must be noted that lifejackets are just one part of a comprehensive drowning prevention strategy and other risk factors such as speed, collisions, and the role of alcohol must be addressed. Alcohol, in particular, has been identified in the reviewed literature as being associated with decreased lifejacket wear. This is of concern, as the involvement of alcohol increases the risk of an accident and the absence of a lifejacket doubles the risk of a fatal outcome [46]. Further research is required to examine factors underpinning the decision to don, or not don, a lifejacket when alcohol is involved.

The discomfort of lifejackets was also found to be negatively associated with lifejacket wear. With the advent of new lifejacket technology, including slim fit and inflatable jackets, the comfort of lifejackets has greatly improved in recent years [47]. Qualitative research should be undertaken with those groups shown to be at an increased risk due to not wearing lifejackets, such as adult males, to determine if new advances in lifejacket design are likely to have an impact on likelihood of lifejacket wear. Consumer education should also be undertaken to make boaters, paddlers, and fishers aware of new styles of lifejackets including the advantages associated with wearability.

Cost and accessibility were also identified as being negatively associated with lifejacket wear. Continued expansion of Australian state government initiatives such as ‘Old4New lifejacket exchange’ are a strategy that should be explored to address such barriers [47]. Education prior to point of sale and the inclusion of lifejackets and other safety equipment at point of sale for boats and watercraft may reduce risk and enhance compliance with legislation.

It was also identified that those who perceive themselves to be intermediate or expert swimmers are less likely to wear a lifejacket. Such overestimation of skill and underestimation of risk is likely to be a key factor in many drowning fatalities; a point supported by previous research investigating risky water-related behaviours [48–52]. Research that compares self-reported to actual swimming ability with attitudes toward wearing lifejackets would be useful. Findings could be used to inform the development of drowning prevention strategies.

### Factors not associated or inconsistent with lifejacket wear

There was no impact on lifejacket wear when considering education, household income, ethnicity, boating ability, confidence in lifejackets, and waterway type. Inconsistent findings were found for weather and water conditions. For many of these variables, it is often challenging to gather data within the coronial system to determine if these factors are implicated in boating and watercraft-related drowning fatalities. Given other drowning prevention research has shown ethnicity and low household income [53] as well as waterway type [54, 55] to be implicated in drownings, qualitative and exposure studies should be conducted to confirm if such variables do actually have an impact on lifejacket wear.

### Interventions

Three studies [14, 15, 20] discussed the evaluation of interventions designed to increase lifejacket wear. The interventions, mandatory regulations, educational marketing campaigns, or a combination of the two, were all found to have improved lifejacket wear rates. Further studies are required on the effectiveness of interventions aimed at increasing lifejacket wear in a range of different audiences, diverse activities, and in a variety of aquatic locations to guide other countries that wish to address lifejacket wear rates, including LMICs.

### Research gaps and future opportunities

With just three studies (15%) evaluating the impact of interventions on lifejacket wear, there is a need for further intervention studies that are designed and implemented based on rigorous behavioural theory and methods, and that evaluate the impact of the intervention on changing motivations toward and actual lifejacket wear. This may be interventions specifically focusing on lifejackets, or broader strategies, such as those focused on boating safety in general, which may have had an impact on lifejacket wear.

This systematic review also highlighted the need for studies examining the impact of compliance on lifejacket wear. Such studies would be valuable to enable comparison between countries or jurisdictions that either legally require and do not legally require the wearing of lifejackets. This would allow an increased understanding of whether regulation drives compliance or if there are jurisdictions with high compliance without the need for regulation.

There are also a range of other aspects related to lifejacket wear that would benefit from further research. These include the impact of different terminology and styles of jacket on wear rates among different cultures; attitudes toward and use of lifejacket as stratified by swimming ability and competence in or on the water (both perceived and real); and lifejacket wear rates as impacted by different types of aquatic activity such as paddling, jet skis, surfing, kite surfing, and stand up paddleboarding. Future research on topics such as these would further enhance understanding of the role of lifejackets in drowning prevention.

This systematic review also identified a lack of research in the published literature with respect to rock fishing and the impact of lifejackets on death and injury as a result of this dangerous activity [56]. Further research is required into the barriers to lifejacket wear among rock fishers as well as evaluation of interventions aimed at increasing lifejacket wear among this at-risk cohort.

Lifejackets are identified as a drowning prevention strategy by the WHO as part of a strategy to ‘set and enforce safe boating, shipping and ferry regulations’ [3]; however, as evidence in this review, there is a dearth of research from LMICs on the issue of lifejacket wear. As LMICs develop and drowning prevention interventions are strengthened, there is a need for the peer-reviewed publication of the outcomes of such interventions to guide countries still yet to act on lifejacket wear.

This systematic review was limited by the examination of peer-reviewed literature in English and German only. There may be additional relevant literature published in the non-peer reviewed literature and in other languages.

## Conclusion

This systematic review has synthesized a range of factors associated with both increased and decreased lifejacket wear that can inform future drowning prevention interventions aimed at behaviour change. Only three studies examined the impact of interventions associated with increasing lifejacket wear. Further research is required to understand the behavioural factors behind individuals’ decisions to wear or not wear lifejackets. This information, combined with the findings of this review, can be incorporated into prevention strategies to further strengthen the evidence base and effectiveness of interventions aimed at preventing drowning.

## Supporting information

S1 Table PRISMA 2009 checklist. (DOC)

S2 Table Supporting information: Extraction by study. (DOCX)
