# Supplementary material for: Personal, social, and environmental factors associated with lifejacket wear in adults and children: A systematic literature review
Source: PLoS One. 2018 May 2;13(5):e0196421. doi: 10.1371/journal.pone.0196421 (PMC5931488; doi:10.1371/journal.pone.0196421)
Supplement: S2 Table — (DOCX) [file pone.0196421.s002.docx]

# S2 Table: Extraction by Study

| **Reference** | ***Bennett et al (1999)*** | | | |
| --- | --- | --- | --- | --- |
| **Factor** | **Outcome (specific)/Group** | | **Result** | **Sig.** |
| Age | Parental age lower than 40 years (awareness of campaign) | | Used lifejackets more than half the time: 69% (n=93)  Used lifejackets less than half the time: 49% (n=164)  OR: 2.3 (95-%-CI: 1.5-3.6) | p<0.001 |
| Confidence in lifejackets | Believe in efficacy of life vest (awareness of campaign) | | Used lifejackets more than half the time: 99% (n=137)  Used lifejackets less than half the time: 99% (n=338)  OR: 1.2 (95-%-CI: 0.1-64.3) | n.s. |
| Comfort of lifejackets | Confident fitting a life vest (awareness of campaign) | | Used lifejackets more than half the time: 94% (n=130)  Used lifejackets less than half the time: 49% (n=164)  OR: 3.2 (95-%-CI: 1.5-7.0) | p=0.003 |
| Education | Not a college graduate (awareness of campaign) | | Used lifejackets more than half the time: 53% (n=72)  Used lifejackets less than half the time: 45% (n=153)  OR: 1.3 (95-%-CI: 0.9-2.0) | n.s. |
| Swimming abilities | Child does not swim well (awareness of campaign) | | Used lifejackets more than half the time: 63% (n=87)  Used lifejackets less than half the time: 52% (n=177)  OR: 1.6 (95-%-CI: 1.1-2.5) | p=0.03 |
| Income | Reported income <$30.000/year (awareness of campaign) | | Used lifejackets more than half the time: 15% (n=17)  Used lifejackets less than half the time: 12% (n=34)  OR: 1.3 (95-%-CI: 0.7-2.6) | n.s. |
| Intervention | Parent recalls campaign | | Used lifejackets more than half the time: 59% (n=81)  Used lifejackets less than half the time: 47% (n=105)  OR: 1.6 (95-%-CI: 1.1-2.5) | p=0.02 |
| Ownership of lifejacket | Child vest ownership (awareness of campaign) | | Used lifejackets more than half the time: 86% (n=199)  Used lifejackets less than half the time: 71% (n=242)  OR: 2.6 (95-%-CI: 1.5-4.4) | <0.001 |
| Perception of danger | Perceived susceptibility of child to drowning (awareness of campaign) | | Used lifejackets more than half the time: 67% (n=92)  Used lifejackets less than half the time: 59% (n=199)  OR: 1.4 (95-%-CI: 0.9-2.1) | n.s. |
| Role modelling | Use of life vest by parents who boat (awareness of campaign) | | Used lifejackets more than half the time: 58% (n=49)  Used lifejackets less than half the time: 48% (n=105)  OR: 1.5 (95-%-CI: 0.9-2.4) | n.s. |
| **Reference** | ***Bugeja et al (2014)*** | | | |
| **Factor** | **Outcome (specific)/Group** | | **Result** | **Sig.** |
| Age | 0 – 29 years of age (Drowning deaths) | | Pre-intervention: n=12  Post-intervention: n=2  Mann-Whitney U-test: 28 | p=0.02 |
|  | 30 – 59 years of age (Drowning deaths) | | Pre-intervention: n=31  Post-intervention: n=10  Mann-Whitney U-test: 27.5 | p=0.02 |
|  | 60+ years of age (Drowning deaths) | | Pre-intervention: n=16  Post-intervention: n=4  Mann-Whitney U-test: 22.0 | n.s. |
| Boat type | Powerboat (<4.8m) (Drowning deaths) | | Pre-intervention: n=25  Post-intervention: n=5  Mann-Whitney U-test:29.5 | p=0.01 |
|  | Powerboat (4.8m-12m) (Drowning deaths) | | Pre-intervention: n=20  Post-intervention: n=6  Mann-Whitney U-test:25.0 | n.s |
|  | Yacht keel boat (Drowning deaths) | | Pre-intervention: n=7  Post-intervention: n=0  Mann-Whitney U-test:27.5 | p=0.02 |
|  | Yacht trailer sailor (Drowning deaths) | | Pre-intervention: n=1  Post-intervention: n=0  Mann-Whitney U-test: n/a | n/a |
|  | Canoe, Kayak, Rowing boat, Raft, Pedal boat or Fun boat (Drowning deaths) | | Pre-intervention: n=3  Post-intervention: n=3  Mann-Whitney U-test:12.5 | n.s |
|  | Kiteboard/Sailboard (Drowning deaths) | | Pre-intervention: n=1  Post-intervention: n=1  Mann-Whitney U-test: n/a | n.s |
|  | PWC (Drowning deaths) | | Pre-intervention: n=2  Post-intervention: n=0  Mann-Whitney U-test: n/a | n.s |
|  | Motorised (Drowning deaths) | | Pre-intervention: n=47  Post-intervention: n=12  Mann-Whitney U-test:29.5 | p=0.01 |
|  | Non-motorised (Sail powered) (Drowning deaths) | | Pre-intervention: n=9  Post-intervention: n=1  Mann-Whitney U-test:26.0 | p=0.04 |
|  | Non-motorised (Human-powered) (Drowning deaths) | | Pre-intervention: n=3  Post-intervention: n=3  Mann-Whitney U-test: n/a | n.s |
| Gender | Male (Drowning deaths) | | Pre-intervention: n=58  Post-intervention: n=16  Mann-Whitney U-test:30.0 | p=0.01 |
|  | Female (Drowning deaths) | | Pre-intervention: n=1  Post-intervention: n=0  Mann-Whitney U-test: n/a | n.s |
| Waterway type | Enclosed (Drowning deaths) | | Pre-intervention: n=21  Post-intervention: n=10  Mann-Whitney U-test:20.5 | n.s |
|  | Coastal (Drowning deaths) | | Pre-intervention: n=17  Post-intervention: n=5  Mann-Whitney U-test:24.0 | n.s |
|  | Inland (Drowning deaths) | | Pre-intervention: n=21  Post-intervention: n=1  Mann-Whitney U-test:30.0 | p=0.01 |
| **Reference** | ***Cassell et al (2015)*** | | | |
| **Factor** | **Outcome (specific)/Group** | | **Result** | **Sig.** |
| Overall | Lifejacket Use (after regulations vs before), all vessel occupants | | Small vessel: OR= 8.173 (95-%-CI: 6.612-1.0103) | p<0.001 |
|  |  |  | Large vessel: OR= 1.267(95-%-CI: 0.943-1.703) | n.s. |
| Age | Lifejacket Use (after regulations vs before), 0 – 9 years of age | | Small vessel: OR=8.776 (95-%-CI: 2.550-30.200) | p<0.001 |
|  |  |  | Large vessel: OR=1.235 (95-%-CI: 0.657-2.231) | n.s. |
|  | Lifejacket Use (after regulations vs before), 10 - 17 years of age | | Small vessel: OR=5.685 (95-%-CI: 3.359-9.621) | <0.001 |
|  |  |  | Large vessel: OR= 1.730 (95-%-CI: 1.006-2.973) | p=0.047 |
|  | Lifejacket Use (after regulations vs before), 18 - 59 years of age | | Small vessel: OR= 7.983 (95-%-CI: 6.236-10.177) | p<0.001 |
|  |  |  | Large vessel: OR= 1.126 (95-%-CI: 0.724-1.752) | n.s. |
|  | Lifejacket Use (after regulations vs before), 60+ years of age | | Small vessel: OR= 33.560 (95-%-CI: 10.893-103.391) | p<0.001 |
|  |  |  | Large vessel: OR= 0.522 (95-%-CI: 0.102-2.673) | n.s. |
| Boat type | Lifejacket Use (after regulations vs before), Open | | Small vessel: OR= 27.705 (95-%-CI: 17.772-43.189) | p<0.001 |
|  |  |  | Large vessel: OR= 2.008 (95-%-CI: 1.035-3.895) | p=0.039 |
|  | Lifejacket Use (after regulations vs before) , Cuddy/half cabin cruiser | | Small vessel: OR= 26.278 (95-%-CI: 12.665-54.522) | p<0.001 |
|  |  |  | Large vessel: OR= 1.168 (95-%-CI: 0.655-2.083) | n.s. |
|  | Lifejacket Use (after regulations vs before) , Full cabin cruiser | | Large vessel: OR= 0.544 (95-%-CI: 0.172-1.718) | n.s. |
|  | Lifejacket Use (after regulations vs before) , Ski boat | | Small vessel: OR= 27.485 (95-%-CI: 4.504-167.716) | <0.001 |
|  |  |  | Large vessel: OR= 1.214 (95-%-CI: 0.645-2.282) | n.s. |
|  | Lifejacket Use (after regulations vs before) , PWC | | Small vessel: OR= 2.648 (95-%-CI: 1.030-6.805) | p=0.043 |
|  | Lifejacket Use (after regulations vs before) , Yacht | | Large vessel: OR= 0.212 (95-%-CI: 0.072-0.626) | p=0.005 |
|  | Lifejacket Use (after regulations vs before) , Other/unknown | | Small vessel: OR= 6.882 (95-%-CI: 2.327-20.352) | p<0.001 |
|  |  |  | Large vessel: OR= 29.503 (95-%-CI: 3.159-275.491) | p=0.003 |
| Gender | Lifejacket Use (after regulations vs before), Males | | Small vessel: OR= 8.223 (95-%-CI: 6.504-10.396) | p<0.001 |
|  |  |  | Large vessel: OR= 1.381 (95-%-CI: 0.951-2.006) | n.s. |
|  | Lifejacket Use (after regulations vs before), Females | | Small vessel: OR= 7.952 (95-%-CI: 4.844-13.054) | p<0.001 |
|  |  |  | Large vessel: OR= 1.096 (95-%-CI: 0.675-1.778) | n.s. |
| Type of activity | Lifejacket Use (after regulations vs before), activity: Fishing | | Small vessel: OR= 60.438 (95-%-CI: 32.715-111.652) | p<0.001 |
|  |  |  | Large vessel: OR= 29.503 (95-%-CI: 3.159-275.491) | p=0.003 |
|  | Lifejacket Use (after regulations vs before) , Towed water sports | | Small vessel: OR= 4.395 (95-%-CI: 1.595-12.111) | p=0.004 |
|  |  |  | Large vessel: OR= 1.775 (95-%-CI: 0.950-3.316) | n.s. |
|  | Lifejacket Use (after regulations vs before) , Pleasure cruising/general | | Small vessel: OR= 18.348 (95-%-CI: 10.471-32.150) | p<0.001 |
|  |  |  | Large vessel: OR= 1.432 (95-%-CI: 0.765-2.681) | n.s. |
|  | Lifejacket Use (after regulations vs before) , PWC riding | | Small vessel: OR= 2.612 (95-%-CI: 1.016-6.171) | p=0.046 |
|  | Lifejacket Use (after regulations vs before) , Sailing | | Large vessel: OR= 0.378 (95-%-CI: 0.143-0.996) | p=0.049 |
|  | Lifejacket Use (after regulations vs before) , Other | | Small vessel: OR= 1.376 (95-%-CI: 0.495-3.822) | n.s. |
|  |  |  | Large vessel: OR= 1.979 (95-%-CI: 0.528-7.422) | n.s. |
| **Reference** | ***Chung et al (2014)*** | | | |
| **Factor** | **Outcome (specific)/Group** | | **Result** | **Sig.** |
| Age | Observed lifejacket use, 0 – 5 years of age | | 89.0% (n=89) | n/a |
|  |  |  | Risk Ratio (bivariate): 4.1 (95-%-CI: 3.2-5.2) | p<0.05 |
|  |  |  | Risk Ratio (multivar., adjusted): 5.0 (95-%-CI: 4.4-5.7) |  |
|  | Observed lifejacket use, 6 – 12 years of age | | 80.0% (n=340) | n/a |
|  |  |  | Risk Ratio (bivariate): 3.7 (95-%-CI: 3.3-4.1) | p<0.05 |
|  |  |  | Risk Ratio (multivar., adjusted): 4.2 (95-%-CI: 3.9-4.6) | p<0.05 |
|  | Observed lifejacket use, 13 – 17 years of age | | 49.5% (n=221) | n/a |
|  |  |  | Risk Ratio (bivariate): 2.3 (95-%-CI: 2.0-2.6) | p<0.05 |
|  |  |  | Risk Ratio (multivar., adjusted): 2.1 (95-%-CI: 1.8-2.3) | p<0.05 |
|  | Observed lifejacket use, 18 – 64 years of age | | 21.8% (n=854) | n/a |
|  |  |  | Risk Ratio (bivariate): reference | n/a |
|  |  |  | Risk Ratio (multivar., adjusted): reference | n/a |
|  | Observed lifejacket use, 65 years of age and older | | 18.3% (n=23) | n/a |
|  |  |  | Risk Ratio (bivariate): 0.8 (95-%-CI: 0.69-1.0) | n.s. |
|  |  |  | Risk Ratio (multivar., adjusted): 1.1 (95-%-CI: 0.84-1.5) | n.s. |
| Boat type | Observed lifejacket use, Motorised boat | | 21.1% (n=887) | n/a |
|  |  |  | Risk Ratio (bivariate): reference | n/a |
|  |  |  | Risk Ratio (multivar., adjusted): reference | n/a |
|  | Observed lifejacket use, PWC | | 96.8% (n=333) | n/a |
|  |  |  | Risk Ratio (bivariate): 4.6 (95-%-CI: 2.7-7.9) | p<0.05 |
|  |  |  | Risk Ratio (multivar., adjusted): 3.7 (95-%-CI: 2.9-4.7) | p<0.05 |
|  | Observed lifejacket use, Kayak | | 79.8% (n=166) | n/a |
|  |  |  | Risk Ratio (bivariate): 3.8 (95-%-CI: 2.5-5.7) | p<0.05 |
|  |  |  | Risk Ratio (multivar., adjusted): 4.4 (95-%-CI: 4.0-4.9) | p<0.05 |
|  | Observed lifejacket use, Canoe | | 59.9% (n=88) | n/a |
|  |  |  | Risk Ratio (bivariate): 2.8 (95-%-CI: 1.6-4.9) | p<0.05 |
|  |  |  | Risk Ratio (multivar., adjusted): 1.8 (95-%-CI: 1.5-2.2) | p<0.05 |
|  | Observed lifejacket use, Rowboat/dinghy | | 45.1% (n=23) | n/a |
|  |  |  | Risk Ratio (bivariate): 2.1 (95-%-CI: 0.93-4.9) | n.s. |
|  |  |  | Risk Ratio (multivar., adjusted): 1.8 (95-%-CI: 1.5-2.2) | p<0.05 |
|  | Observed lifejacket use, Paddleboard/sailboard | | 62.0% (n=31) | n/a |
|  |  |  | Risk Ratio (bivariate): 2.9 (95-%-CI: 1.9-4.6) | p<0.05 |
|  |  |  | Risk Ratio (multivar., adjusted): 2.2 (95-%-CI: 2.1-2.3) | p<0.05 |
|  | Observed lifejacket use, Sailboat | | 31.7% (n=39) | n/a |
|  |  |  | Risk Ratio (bivariate): 1.5 (95-%-CI: 1.4-1.6) | p<0.05 |
|  |  |  | Risk Ratio (multivar., adjusted): 2.3 (95-%-CI: 2.3-2.3) | p<0.05 |
|  | Observed lifejacket use, Inflatable/raft | | 43.3% (n=26) | n/a |
|  |  |  | Risk Ratio (bivariate): 2.1 (95-%-CI: 1.5-2.7) | p<0.05 |
|  |  |  | Risk Ratio (multivar., adjusted): 1.2 (95-%-CI: 0.57-2.4) | n.s. |
| Gender | Observed lifejacket use, Male | | 27.8% (n=899) | n/a |
|  |  |  | Risk Ratio (bivariate): reference | n/a |
|  | Observed lifejacket use, Female | | 33.8% (n=618) | n/a |
|  |  |  | Risk Ratio (bivariate): 1.3 (95-%-CI: 1.1-1.5) | p<0.05 |
| Role modelling | Observed lifejacket use, likelihood of child (0 – 5 years) lifejacket use if any adult in the boat worse a lifejacket | | Multivariable incidence risk ratio: 6.6 (0.7-63.4) | n.s. |
|  | Observed lifejacket use, likelihood of child (6 - 12 years) lifejacket use if any adult in the boat worse a lifejacket | | Multivariable incidence risk ratio: 6.2 (0.5-83) | n.s. |
|  | Observed lifejacket use, likelihood of child (13 - 17 years) lifejacket use if any adult in the boat worse a lifejacket | | Multivariable incidence risk ratio: 20.0 (2.9-135) | p<0.05 |
| Type of activity | Observed lifejacket use, Fishing/intent to fish | | 21.2% (n=191) | n/a |
|  |  |  | Risk Ratio (bivariate): reference | n/a |
|  |  |  | Risk Ratio (multivar., adjusted): reference | n/a |
|  | Observed lifejacket use, Water-skiing | | 44.3% (n=214) | n/a |
|  |  |  | Risk Ratio (bivariate): 2.1 (95-%-CI: 1.4-3.1) | p<0.05 |
|  |  |  | Risk Ratio (multivar., adjusted): 1.5 (95-%-CI: 1.1-2.0) | p<0.05 |
|  | Observed lifejacket use, Racing/high-speed | | 56.9% (n=111) | n/a |
|  |  |  | Risk Ratio (bivariate): 2.7 (95-%-CI: 2.6-2.8) | p<0.05 |
|  |  |  | Risk Ratio (multivar., adjusted): 1.0 (95-%-CI: 0.81-1.3) | n.s. |
|  | Observed lifejacket use, Swimming | | 50.0% (n=8) | n/a |
|  |  |  | Risk Ratio (bivariate): 2.4(95-%-CI: 1.5-3.8) | p<0.05 |
|  |  |  | Risk Ratio (multivar., adjusted): 2.1 (95-%-CI: 0.88-5.2) | n.s. |
|  | Observed lifejacket use, Pleasure | | 29.8% (n=1038) | n/a |
|  |  |  | Risk Ratio (bivariate): 1.4 (95-%-CI: 1.0-2.0) | p<0.05 |
|  |  |  | Risk Ratio (multivar., adjusted): 2.1 (95-%-CI: 0.88-5.2) | n.s. |
|  | Observed lifejacket use, Other activity | | 72.7% (n=10) | n/a |
|  |  |  | Risk Ratio (bivariate): 1.3 (95-%-CI: 0.3-5.7) | n.s. |
|  |  |  | Risk Ratio (multivar., adjusted): 0.95 (95-%-CI: 0.27-3.4) | n.s. |
| Weather/Water conditions | Observed lifejacket use, Sunny | | 32.7% (n=765) | n/a |
|  |  |  | Risk Ratio (bivariate): reference (for weather cond.) | n/a |
|  |  |  | Risk Ratio (multivar., adjusted): reference (for weather cond.) | n/a |
|  | Observed lifejacket use, Partly cloudy | | 29.3% (n=591) | n/a |
|  |  |  | Risk Ratio (bivariate): 0.9 (95-%-CI: 0.84-0.96) | p<0.05 |
|  |  |  | Risk Ratio (multivar., adjusted): 0.95 (95-%-CI: 0.94-0.97) | p<0.05 |
|  | Observed lifejacket use, Cloudy | | 32.7% (n=219) | n/a |
|  |  |  | Risk Ratio (bivariate): 1.0 (95-%-CI: 0.97-1.0) | n.s. |
|  |  |  | Risk Ratio (multivar., adjusted): 1.0 (95-%-CI: 0.9-1.1) | n.s. |
|  | Observed lifejacket use, Raining | | 7.8% (n=10) | n/a |
|  |  |  | Risk Ratio (bivariate): 0.2 (95-%-CI: 0.15-0.38) | p<0.05 |
|  |  |  | Risk Ratio (multivar., adjusted): 0.39 (95-%-CI: 0.33-0.46) | p<0.05 |
|  | Observed lifejacket use, Not choppy (6) | | 29.9% (n=1405) | n/a |
|  |  |  | Risk Ratio (bivariate): reference (for water cond.) | n/a |
|  |  |  | Risk Ratio (multivar., adjusted): reference (for water cond.) | n/a |
|  | Observed lifejacket use, Choppy (waves above 6 inches) | | 38.4% (n=180) | n/a |
|  |  |  | Risk Ratio (bivariate): 1.2 (95-%-CI: 0.7-2.4) | n.s. |
|  |  |  | Risk Ratio (multivar., adjusted): 1.3 (95-%-CI: 1.0-1.7) | p<0.05 |
| **Reference** | ***Clemens et al (2016)*** | | | |
| **Factor** | **Outcome (specific)/Group** | **Result** | | **Sig.** |
| Age | Lifejacket use (drowning deaths), All ages | Lifejacket worn: 16.4% (n=121); water-related fatalities per 100,000 population/year: 0.07  Lifejacket not worn: 65.6% (n=485); water-related fatalities per 100,000 population/year: 0.29 | | n.g. |
|  | Lifejacket use (drowning deaths), 5 – 14 years of age | Lifejacket worn: 46.2% (n=6); water-related fatalities per 100,000 population/year: 0.03  Lifejacket not worn: 53.8% (n=7); water-related fatalities per 100,000 population/year: 0.04 | | n.g. |
|  | Lifejacket use (drowning deaths), 15 – 19 years of age | Lifejacket worn: 8.0% (n=4); water-related fatalities per 100,000 population/year: 0.04  Lifejacket not worn: 43% (n=86); water-related fatalities per 100,000 population/year: 0.38 | | n.g. |
|  | Lifejacket use (drowning deaths), 20 – 34 years of age | Lifejacket worn: 11.1% (n=21); water-related fatalities per 100,000 population/year: 0.06  Lifejacket not worn: 86.4% (n=130); water-related fatalities per 100,000 population/year: 0.37 | | n.g. |
|  | Lifejacket use (drowning deaths), 35 – 64 years of age | Lifejacket worn: 19.2% (n=72); water-related fatalities per 100,000 population/year: 0.10  Lifejacket not worn: 61.6% (n=231); water-related fatalities per 100,000 population/year: 0.32 | | n.g. |
|  | Lifejacket use (drowning deaths), 65 years of age or older | Lifejacket worn: 16.2% (n=18); water-related fatalities per 100,000 population/year: 0.07  Lifejacket not worn: 66.7% (n=74); water-related fatalities per 100,000 population/year: 0.31 | | n.g. |
| **Reference** | ***Croft et al (2015)*** | | | |
| **Factor** | **Outcome (specific)/Group** | | **Result** | **Sig.** |
| Alcohol and other drugs | Lifejacket use and availability, No alcohol consumed | | Lifejacket available but not worn: n=52  Lifejacket not available: n=103  Lifejacket worn: n=52 | n.g. |
|  | Lifejacket use and availability, alcohol consumed | | Lifejacket available but not worn: n=18  Lifejacket not available: n=21  Lifejacket worn: n=unclear* | n.g. |
| **Reference** | ***Dai et al (2013)*** | | | |
| **Factor** | **Outcome (specific)/Group** | | **Result** | **Sig.** |
| Race/Ethnicity | Lifejacket worn | | Total: 1.36%(n=3)  White/Caucasian: 0.21 per 100,000 children  Black: 0.00 per 100,000 children  Rate ratio: 0 (95-%-CI: 0-4.13) | n.s. |
|  | Lifejacket not worn | | Total: 92.27%(n=203)  White/Caucasian: 8.65 per 100,000 children  Black: 7.86 per 100,000 children  Rate ratio: 0.91 (95-%-CI: 0.66-1.24) | n.s. |
| **Reference** | ***Giles et al (2010)*** | | | |
| **Factor** | **Outcome** | | | |
| Accessibility of lifejackets | Lifejackets are perceived to be inaccessible, particularly in regional and remote areas, driving up prices. | | | |
| Boating abilities | Participants in this study perceived the use of lifejacket as optional and related to the skill level and experience of boaters. Lifejackets are perceived to be of higher importance to those of lower skill levels. | | | |
| Confidence in lifejackets | Lifejackets are overall perceived to be ineffective, particularly in cold water, and as such deemed unnecessary. | | | |
| Role modelling | Elders in the First Nation community are of high value and perceived to be role models. Elders often do not wear lifejackets and function as role models for younger people who listen to their advice and refuse to wear lifejackets as well. | | | |
| **Reference** | ***Jones (1999)*** | | | |
| **Factor** | **Outcome (specific)/Group** | | **Result** | **Sig.** |
| Seat position | Lifejacket use by seat position on PWCs | | Operator: 98% (n=181)  Rider: 98% (n=56) | n.s. |
| **Reference** | ***Mangione et al (2012)*** | | | |
| **Factor** | **Outcome (specific)/Group** | | **Result** | **Sig.** |
| Age | Lifejacket use 1999/2010, 0 - 5 years of age | | 1999: 81.1% (N=493); 2010: 94.8% (N =804) | n.g. |
|  | Lifejacket use 1999/2010, 6 - 12 years of age | | 1999: 68.3% (N =1987); 2010: 87.9% (N =2545) | n.g. |
|  | Lifejacket use 1999/2010, 13 - 17 years of age | | 1999: 22.0% (N =1977); 2010: 29.0% (N =1947) | n.g. |
|  | Lifejacket use 1999/2010, 18 - 64 years of age | | 1999: 7.3% (N =24072); 2010: 7.3% (N =36070) | n.g. |
|  | Lifejacket use 1999/2010, 65 years of age and older | | 1999: 11.6% (N =1140); 2010: 10.7% (N =763) | n.g. |
|  | Lifejacket use 1999/2010, All | | 1999: 14.3% (N =29669); 2010: 15.6% (N =42129) | n.g. |
|  | Lifejacket use 1999/2010, All youth | | 1999: 51.1% (N =4457); 2010: 64.9% (N =5296) | n.g. |
|  | Lifejacket use 1999/2010, All adults | | 1999: 7.5% (N =25212); 2010: 7.4% (N =36833) | n.g. |
|  | Lifejacket use 1999 - 2010, 0 - 5 years of age | | 94.3% (N =9087); z-score: 8.101 | p<0.0001 |
|  | Lifejacket use 1999 - 2010, 6 - 12 years of age | | 79.7% (N =30309); z-score: 22.199 | p<0.0001 |
|  | Lifejacket use 1999 - 2010, 13 - 17 years of age | | 27.9% (N =28069); z-score: 6.494 | p<0.0001 |
|  | Lifejacket use 1999 - 2010, 18 - 64 years of age | | 8.1% (N =368192); z-score: -0.880 | n.s. |
|  | Lifejacket use 1999 - 2010, 65 years of age and older | | 8.5% (N =12394); z-score: -0.829 | n.s. |
|  | Lifejacket use 1999 - 2010, All | | 15.9% (N =448051); z-score: 6.471 | p<0.0001 |
|  | Lifejacket use 1999 - 2010, All youth | | 59.8% (N =67465); z-score: 16.573 | p<0.0001 |
|  | Lifejacket use 1999 - 2010, All adults | | 8.1% (N =380586); z-score: -1.009 | n.s. |
| Boat type | Lifejacket use 1999/2010, All power boats (no PWCs) | | 1999: 4.0% (N =19789); 2010: 3.7% (N =20727) | n.g. |
|  | Lifejacket use 1999/2010, Skiff/Utility | | 1999: 9.8% (N =1867); 2010: 9.6% (N =6628) | n.g. |
|  | Lifejacket use 1999/2010, Runabout/Speedboat | | 1999: 3.6% (N =13096); 2010: 2.2% (N =14947) | n.g. |
|  | Lifejacket use 1999/2010, Cabin cruiser | | 1999: 1.7% (N =3394); 2010: 1.5% (N =5898) | n.g. |
|  | Lifejacket use 1999/2010, House boat | | 1999: 0.0% (N =151); 2010: 0.0% (N =140) | n.g. |
|  | Lifejacket use 1999/2010, Pontoon | | 1999: 3.6% (N =1228); 2010: 1.1% (N =2909) | n.g. |
|  | Lifejacket use 1999/2010, Powered inflatable | | 1999: 15.9% (N =204); 2010: 16.7% (N =345) | n.g. |
|  | Lifejacket use 1999 - 2010, All power boats (no PWCs) | | 3.9% (N =313179); z-score: -4.499 | p<0.0001 |
|  | Lifejacket use 1999 - 2010, Skiff/Utility | | 8.3% (N =53006); z-score: -1.211 | n.s. |
|  | Lifejacket use 1999 - 2010, Runabout/Speedboat | | 3.3% (N =173082); z-score: -13.240 | p<0.0001 |
|  | Lifejacket use 1999 - 2010, Cabin cruiser | | 1.5% (N =61802); z-score: 0.103 | n.s. |
|  | Lifejacket use 1999 - 2010, House boat | | 0.8% (N =1795); z-score: 0.296 | n.s. |
|  | Lifejacket use 1999 - 2010, Pontoon | | 2.4% (N =23436); z-score: -7.982 | p<0.0001 |
|  | Lifejacket use 1999 - 2010, Powered inflatable | | 15.3% (N =2853); z-score: -1.230 | n.s. |
|  | Lifejacket use 1999/2010, All sail craft | | 1999: 13.4% (N =3417); 2010: 22.0% (N =3336) | n.g. |
|  | Lifejacket use 1999/2010, Sailboard | | 1999: 16.9% (N =46); 2010: 83.2% (N =29) | n.g. |
|  | Lifejacket use 1999/2010, Day Sailor | | 1999: 30.7% (N =738); 2010: 57.5% (N =731) | n.g. |
|  | Lifejacket use 1999/2010, Cabin sailboat | | 1999: 9.2% (N =2633); 2010: 11.7% (N =2576) | n.g. |
|  | Lifejacket use 1999/2010, All paddled craft | | 1999: 45.3% (N =1676); 2010: 35.4% (N =2548) | n.g. |
|  | Lifejacket use 1999/2010, Paddled inflatable | | 1999: 71.3% (N =174); 2010: 7.0% (N =812) | n.g. |
|  | Lifejacket use 1999/2010, Rowboat/Dinghy | | 1999: 24.7% (N =82); 2010: 34.8% (N =46) | n.g. |
|  | Lifejacket use 1999/2010, Canoe | | 1999: 17.6% (N =809); 2010: 19.0% (N =993) | n.g. |
|  | Lifejacket use 1999/2010, Kayak | | 1999: 82.7% (N =611); 2010: 76.0% (N =697) | n.g. |
|  | Lifejacket use 1999 - 2010, All sail craft | | 20.2% (N =41941); z-score: 15.246 | p<0.0001 |
|  | Lifejacket use 1999 - 2010, Sailboard | | 76.7% (N =305); z-score: 5.144 | p<0.0001 |
|  | Lifejacket use 1999 - 2010, Day Sailor | | 47.3% (N =8835); z-score: 15.289 | p<0.0001 |
|  | Lifejacket use 1999 - 2010, Cabin sailboat | | 12.3% (N =32801); z-score: 8.532 | p<0.001 |
|  | Lifejacket use 1999 - 2010, All paddled craft | | 47.1% (N =21473); z-score: -12.17 | p<0.0001 |
|  | Lifejacket use 1999 - 2010, Paddled inflatable | | 39.4% (N =4023); z-score: -25.584 | p<0.0001 |
|  | Lifejacket use 1999 - 2010, Rowboat/Dinghy | | 25.6% (N =1041); z-score: -0.080 | n.s. |
|  | Lifejacket use 1999 - 2010, Canoe | | 22.4% (N =8243); z-score: -2.440 | p=0.0147 |
|  | Lifejacket use 1999 - 2010, Kayak | | 78.6% (N =8166); z-score: -11.008 | p<0.0001 |
| **Reference** | ***Mangione et al (2014)*** | | | |
| **Factor** | **Outcome (specific)/Group** | | **Result** | **Sig.** |
| Age | Lifejacket use, Children ages 0 – 12 years (intervention site; comparison site) | | California (baseline): 80.8% (N=299); 77.7% (N=273)  California (post-int., year 1): 77.3% (N=482); 71.1% (N=278)  California (post-int., year 2): 87.5% (N=1797); 85.3% (N=654)  California (post-int., year 3): 91.2% (N=1699); 87.8% (N=342)  Mississippi (baseline): 95.4% (N=293); 96.5% (N=88)  Mississippi (post-int., year 1): 96.5% (N=909); 84.1% (N=623)  Mississippi (post-int., year 2): 97.6% (N=758); 88.6% (N=708)  Mississippi (post-int., year 3): 95.2% (N=856); 86.4% (N=686) | n.g. |
|  | Lifejacket use, Teenager ages 13 – 17 years (intervention site; comparison site) | | California (baseline): 15.9% (N=218); 15.9% (N=182)  California (post-int., year 1): 14.7% (N=529); 18.2% (N=231)  California (post-int., year 2): 21.2% (N=1484); 22.4% (N=513)  California (post-int., year 3): 26.8% (N=1124); 32.6% (N=184)  Mississippi (baseline): 37.9% (N=139); 29.0% (N=63)  Mississippi (post-int., year 1): 88.0% (N=686); 37.6% (N=330)  Mississippi (post-int., year 2): 87.8% (N=509); 29.1% (N=319)  Mississippi (post-int., year 3): 88.7% (N=435); 30.3% (N=318) | n.g. |
|  | Lifejacket use, Adults (intervention site; comparison site) | | California (baseline): 8.5% (N=3138); 3.9% (N=2551)  California (post-int., year 1): 12.1% (N=5296); 3.9% (N=)  California (post-int., year 2): 9.4% (N=16189); 5.5% (N=4277)  California (post-int., year 3): 10.5% (N=17466); 5.7% (N=4035)  Mississippi (baseline): 13.7% (N=2200); 7.2% (N=888)  Mississippi (post-int., year 1): 75.6% (N=5854); 8.9% (N=4655)  Mississippi (post-int., year 2): 70.1% (N=6399); 9.3% (N=5480)  Mississippi (post-int., year 3): 68.1% (N=6128); 8.2% (N=4295) | n.g. |
| Boat type | Lifejacket use, Boat size under 16 feet (intervention site; comparison site) | | California (baseline): 3.8% (N=122); 4.7% (N=427)  California (post-int., year 1): 17.6% (N=165); 4.6% (N=327)  California (post-int., year 2): 16.9% (N=621); 11.1% (N=286)  California (post-int., year 3): 17.1% (N=1159); 9.6% (N=810)  Mississippi (baseline): 18.6% (N=204); 11.5% (N=107)  Mississippi (post-int., year 1): 72.2% (N=765); 17.9% (N=319)  Mississippi (post-int., year 2): 65.1% (N=608); 15.1% (N=219)  Mississippi (post-int., year 3): 56.8% (N=869); 13.8% (N=177) | n.g. |
|  | Lifejacket use, Boat size: 16 – 21 feet (intervention site; comparison site) | | California (baseline): 10.8% (N=1840); 3.4% (N=1415)  California (post-int., year 1): 13.7% (N=3788); 3.2% (N=1248)  California (post-int., year 2): 11.2% (N=8569); 6.1% (N=2596)  California (post-int., year 3): 13.9% (N=8882); 6.0% (N=1615)  Mississippi (baseline): 17.3% (N=1522); 9.1% (N=594)  Mississippi (post-int., year 1): 79.0% (N=2734); 13.0% (N=2233)  Mississippi (post-int., year 2): 76.9% (N=3424); 13.2% (N=2691)  Mississippi (post-int., year 3): 74.4% (N=3204); 11.2% (N=2151) | n.g. |
|  | Lifejacket use, Boat size: 21 – 26 feet (intervention site; comparison site) | | California (baseline): 4.4% (N=568); 4.0% (N=487)  California (post-int., year 1): 7.5% (N=841); 6.4% (N=345)  California (post-int., year 2): 7.1% (N=5558); 4.8% (N=1158)  California (post-int., year 3): 4.8% (N=5606); 4.0% (N=895)  Mississippi (baseline): 7.2% (N=474); 4.7% (N=187)  Mississippi (post-int., year 1): 72.8% (N=2355); 3.2% (N=2103)  Mississippi (post-int., year 2): 63.8% (N=2367); 3.7% (N=2570)  Mississippi (post-int., year 3): 64.5% (N=2055); 3.7% (N=1967) | n.g. |
| Gender | Lifejacket use, Adult males (intervention site; comparison site) | | California (baseline): 11.1% (N=2062); 4.3% (N=1790)  California (post-int., year 1): 15.1% (N=3625); 4.0% (N=1648)  California (post-int., year 2): 12.3% (N=10432); 5.6% (N=2725)  California (post-int., year 3): 14.1% (N=10701); 5.9% (N=2714)  Mississippi (baseline): 15.9% (N=1524); 8.3% (N=676)  Mississippi (post-int., year 1): 77.2% (N=4111); 11.6% (N=2952)  Mississippi (post-int., year 2): 71.1% (N=4435); 11.9% (N=3340)  Mississippi (post-int., year 3): 68.0% (N=4359); 10.8% (N=2581) | n.g. |
|  | Lifejacket use, Adult females (intervention site; comparison site) | | California (baseline): 2.7% (N=1073); 2.7% (N=757)  California (post-int., year 1): 5.6% (N=1660); 3.6% (N=643)  California (post-int., year 2): 3.4% (N=5750); 5.3% (N=1551)  California (post-int., year 3): 3.3% (N=6755); 5.2% (N=1321)  Mississippi (baseline): 8.4% (N=675); 5.2% (N=212)  Mississippi (post-int., year 1): 72.1% (N=1737); 4.3% (N=1702)  Mississippi (post-int., year 2): 67.6% (N=1954); 4.5% (N=2131)  Mississippi (post-int., year 3): 68.4% (N=1769); 3.6% (N=1714) | n.g. |
| Type of activity | Lifejacket use, Fishing (intervention site; comparison site) | | California (baseline): 26.2% (N=365); 5.9% (N=524)  California (post-int., year 1): 36.5% (N=1233); 3.4% (N=619)  California (post-int., year 2): 30.5% (N=36.6); 8.6% (N=629)  California (post-int., year 3): 36.6% (N=1429); 8.6% (N=629)  Mississippi (baseline): 26.2% (N=652); 25.3% (N=409)  Mississippi (post-int., year 1): 84.4% (N=2147); 30.6% (N=964)  Mississippi (post-int., year 2): 77.4% (N=2137); 30.0% (N=724)  Mississippi (post-int., year 3): 72.3% (N=2509); 28.7% (N=581) | n.g. |
|  | Lifejacket use, Other activities (intervention site; comparison site) | | California (baseline): 3.2% (N=2782); 3.2% (N=2027)  California (post-int., year 1): 4.7% (N=4053); 4.0% (N=1673)  California (post-int., year 2): 2.9% (N=13692); 4.3% (N=3721)  California (post-int., year 3): 2.6% (N=16037); 4.6% (N=3406)  Mississippi (baseline): 6.5% (N=1548); 2.6% (N=479)  Mississippi (post-int., year 1): 70.6% (N=3707); 3.3% (N=3691)  Mississippi (post-int., year 2): 65.9% (N=4262); 3.8% (N=4756)  Mississippi (post-int., year 3): 65.7% (N=3619); 2.8% (N=3714) | n.g. |
| **Reference** | ***Moran (2011)*** | | | |
| **Factor** | **Outcome (specific)/Group** | | **Result** | **Sig.** |
| Gender | Reported seeing their friends not wearing a lifejacket | | Males: 72.2%  Females: 59.8%  χ^2^ = 30.479 | p<0.001 |
| **Reference** | ***Nathanson et al (2010)*** | | | |
| **Factor** | **Outcome (specific)/Group** | | **Result** | **Sig.** |
| Age | Lifejacket use | | Use of lifejackets among sailors under 30 years of age is significantly higher than among those over the age of 30 (χ^2^ = 9.72)^$^ | p<0.01 |
| **Reference** | ***Quan et al (1998)*** | | | |
| **Factor** | **Outcome (specific)/Group** | | **Result** | **Sig.** |
| Age | Lifejacket use, all | | 25.4% (N=4181) | n/a |
|  | Lifejacket use, 0 – 4 years | | 90.7% (N=107), Relative Prevalence: 6.7 (95-%-CI: 6.0-7.5) | p<0.05 |
|  | Lifejacket use, 5 – 14 years | | 63.5% (N=840), Relative Prevalence: 5.3 (95-%-CI: 4.7-5.9) | p<0.05 |
|  | Lifejacket use, 14 years and older | | 13.2% (N=3210), Relative Prevalence: reference | n/a |
| Boat type | Lifejacket use, Sailboat | | 50.0% (N=114), Relative Prevalence: 2.3 (95-%-CI: 2.0-2.8) | p<0.05 |
|  | Lifejacket use, Rowboat | | 27.0% (N=400), Relative Prevalence: 1.3 (95-%-CI: 1.1-1.5) | p<0.05 |
|  | Lifejacket use, Canoe | | 40.9% (N=396), Relative Prevalence: 2.2 (95-%-CI: 2.0-2.5) | n.s. |
|  | Lifejacket use, Kayak | | 77.6% (N=107), Relative Prevalence: 5.5 (95-%-CI: 4.9-6.2) | p<0.05 |
|  | Lifejacket use, Raft | | 31.3% (N=96), Relative Prevalence: 1.1 (95-%-CI: 0.8-1.5) | p<0.05 |
|  | Lifejacket use, Motorboat | | 19.0% (N=2640), Relative Prevalence: reference | n/a |
| Gender | Lifejacket use, Female | | 31.4% (N=1450), Relative Prevalence: 1.5 (95-%-CI: 1.3-1.6) | p<0.05 |
|  | Lifejacket use, Male | | 21.2% (N=2686), Relative Prevalence: reference | n/a |
| Water/Weather conditions | Lifejacket use, Cloudy | | 20.6% (N=102), Relative Prevalence: 0.8 (95-%-CI: 0.9-1.3) | n.s. |
|  | Lifejacket use, Partly cloudy | | 29.6% (N=598), Relative Prevalence: 1.2 (95-%-CI: 1.0-1.4) | n.s. |
|  | Lifejacket use, Sunny | | 24.8% (N=3389), Relative Prevalence: reference | n/a |
|  | Lifejacket use, Temperature below 65F | | 26.5% (N=769), Relative Prevalence: 1.0 (95-%-CI: 0.8-1.4) | n.s. |
|  | Lifejacket use, Temperature between 65F and 80F | | 25.4% (N=3068), Relative Prevalence: 1.0 (95-%-CI: 0.9-1.2) | n.s. |
|  | Lifejacket use, Temperature above 80F | | 21.8% (N=285), Relative Prevalence: reference | n/a |
| **Reference** | ***Quistberg et al (2014a)*** | | | |
| **Factor** | **Outcome (specific)/Group** | | **Result^#^** | **Sig.** |
| Accessibility of lifejackets | Lifejacket use, Inflatable lifejacket on board | | High use (51-100%): 37%; Low use (0-50%): 15%  Risk Ratio: 0.78 (95-%-CI: 0.68-0.88) | p<0.05 |
| Age | Lifejacket use, younger than 30 years | | High use (51-100%): 8%; Low use (0-50%): 11%  Risk Ratio: reference | n/a |
|  | Lifejacket use, 30 – 39 years | | High use (51-100%): 14%; Low use (0-50%): 18%  Risk Ratio: 0.99 (95-%-CI: 0.88-1.10) | n.s. |
|  | Lifejacket use, 40 – 49 years | | High use (51-100%): 30%; Low use (0-50%): 33%  Risk Ratio: 0.97 (95-%-CI: 0.88-1.08) | n.s. |
|  | Lifejacket use, 50 – 59 years | | High use (51-100%): 36%; Low use (0-50%): 25%  Risk Ratio: 0.89 (95-%-CI: 0.80-1.00) | n.s. |
|  | Lifejacket use, 60 years and older | | High use (51-100%): 12%; Low use (0-50%): 14%  Risk Ratio: 0.97 (95-%-CI: 0.89-1.09) | n.s. |
| Alcohol and other drugs | Lifejacket use, After drinking | | High use (51-100%): 26%; Low use (0-50%): 16%  Risk Ratio: 0.89 (95-%-CI: 0.80-0.99) | p<0.05 |
| Boat type | Lifejacket use, Boat length: less than 16ft | | High use (51-100%): 20%; Low use (0-50%): 4%  Risk Ratio: reference | n/a |
|  | Lifejacket use, Boat length: 16 – 20ft | | High use (51-100%): 62%; Low use (0-50%): 61%  Risk Ratio: 1.59 (95-%-CI: 1.21-2.08) | p<0.05 |
|  | Lifejacket use, Boat length: 21 – 25.5ft | | High use (51-100%): 18%; Low use (0-50%): 34%  Risk Ratio: 1.71 (95-%-CI: 1.30-2.24) | p<0.05 |
| Boating abilities | Lifejacket use, Piloted today | | High use (51-100%): 81%; Low use (0-50%): 79%  Risk Ratio: 0.99 (95-%-CI: 0.87-1.11) | n.s. |
|  | Lifejacket use, Taken boating safety class | | High use (51-100%): 46%; Low use (0-50%): 36%  Risk Ratio: 0.94 (95-%-CI: 0.87-1.01) | n.s. |
|  | Lifejacket use, Have State boater education card | | High use (51-100%): 17%; Low use (0-50%): 10%  Risk Ratio: 0.90 (95-%-CI: 0.78-1.03) | n.s. |
|  | Lifejacket use, Boat ownership | | High use (51-100%): 84%; Low use (0-50%): 87%  Risk Ratio: 1.05 (95-%-CI: 0.92-1.20) | n.s. |
|  | Lifejacket use, Boating experience: less than 5 years | | High use (51-100%): 10%; Low use (0-50%): 11%  Risk Ratio: reference | n/a |
|  | Lifejacket use, Boating experience: 5 - 9 years | | High use (51-100%): 10%; Low use (0-50%): 7%  Risk Ratio: 0.92 (95-%-CI: 0.78-1.10) | n.s. |
|  | Lifejacket use, Boating experience: 10 - 19 years | | High use (51-100%): 15%; Low use (0-50%): 17%  Risk Ratio: 1.02(95-%-CI: 0.90-1.15) | n.s. |
|  | Lifejacket use, Boating experience: 20 - 29 years | | High use (51-100%): 18%; Low use (0-50%): 22%  Risk Ratio: 1.04 (95-%-CI: 0.93-1.17) | n.s. |
|  | Lifejacket use, Boating experience: 30 - 39 years | | High use (51-100%): 25%; Low use (0-50%): 20%  Risk Ratio: 0.98 (95-%-CI: 0.86-1.12) | n.s. |
|  | Lifejacket use, Boating experience: more than 40 years | | High use (51-100%): 22%; Low use (0-50%): 23%  Risk Ratio: 1.06 (95-%-CI: 0.93-1.21) | n.s. |
|  | Lifejacket use, Times boating last 12 months: less than 6 times | | High use (51-100%): 23%; Low use (0-50%): 14%  Risk Ratio: reference | n/a |
|  | Lifejacket use, Times boating last 12 months: 6 - 10 times | | High use (51-100%): 20%; Low use (0-50%): 19%  Risk Ratio: 1.09 (95-%-CI: 0.95-1.24) | n.s. |
|  | Lifejacket use, Times boating last 12 months: 11 - 20 times | | High use (51-100%): 21%; Low use (0-50%): 31%  Risk Ratio: 1.14 (95-%-CI: 1.00-1.29) | n.s. |
|  | Lifejacket use, Times boating last 12 months: 21 - 30 times | | High use (51-100%): 14%; Low use (0-50%): 17%  Risk Ratio: 1.14 (95-%-CI: 1.00-1.30) | n.s. |
|  | Lifejacket use, Times boating last 12 months: 31 - 40 times | | High use (51-100%): 7%; Low use (0-50%): 5%  Risk Ratio: 1.04 (95-%-CI: 0.86-1.27) | n.s. |
|  | Lifejacket use, Times boating last 12 months: more than 41 times | | High use (51-100%): 17%; Low use (0-50%): 15%  Risk Ratio: 1.09 (95-%-CI: 0.95-1.26) | n.s. |
|  | Lifejacket use, Self-rated piloting skills: Beginner | | High use (51-100%): 3%; Low use (0-50%): 6%  Risk Ratio: 1.09 (95-%-CI: 0.95-1.26) | n.s. |
|  | Lifejacket use, Self-rated piloting skills: Intermediate | | High use (51-100%): 48%; Low use (0-50%): 50%  Risk Ratio: 1.01 (95-%-CI: 0.95-1.09) | n.s. |
|  | Lifejacket use, Self-rated piloting skills: Expert | | High use (51-100%): 49%; Low use (0-50%): 44%  Risk Ratio: reference | n/a |
| Comfort of lifejackets | Lifejacket use, Very comfortable | | High use (51-100%): 75%; Low use (0-50%): 46%  Risk Ratio: reference | n/a |
|  | Lifejacket use, Somewhat comfortable | | High use (51-100%): 21%; Low use (0-50%): 34%  Risk Ratio: 1.19 (95-%-CI: 1.04-1.35) | p<0.05 |
|  | Lifejacket use, Uncomfortable | | High use (51-100%): 4%; Low use (0-50%): 20%  Risk Ratio: 1.29 (95-%-CI: 1.09-1.52) | p<0.05 |
| Confidence in lifejackets | Lifejacket use, Confidence that lifejackets may save from drowning: very confident | | High use (51-100%): 88%; Low use (0-50%): 84%  Risk Ratio: reference | n/a |
|  | Lifejacket use, Confidence that lifejackets may save from drowning: confident | | High use (51-100%): 11%; Low use (0-50%): 14%  Risk Ratio: 1.03 (95-%-CI: 0.94-1.14) | n.s. |
|  | Lifejacket use, Confidence that lifejackets may save from drowning: not confident | | High use (51-100%): 1%; Low use (0-50%): 3%  Risk Ratio: 1.13 (95-%-CI: 0.96-1.32) | n.s. |
| Education | Lifejacket use, Some college education | | High use (51-100%): 79%; Low use (0-50%): 77%  Risk Ratio: 0.98 (95-%-CI: 0.91-1.06) | n.s. |
| Gender | Lifejacket use, Female | | High use (51-100%): 9%; Low use (0-50%): 17%  Risk Ratio: 1.09 (95-%-CI: 1.01-1.17) | p<0.05 |
| Income | Lifejacket use, Median household income (US$) | | High use (51-100%): $76657; Low use (0-50%): $76315  Risk Ratio: 1.00(95-%-CI: 0.99-1.01) | n.s. |
| Role modelling | Lifejacket use, Any children ( less than 10 years) on board | | High use (51-100%): 15%; Low use (0-50%): 12%  Risk Ratio: 0.88 (95-%-CI: 0.79-0.99) | p<0.05 |
|  | Lifejacket use, Any preteens/teens on board | | High use (51-100%): 15%; Low use (0-50%): 14%  Risk Ratio: 0.91 (95-%-CI: 0.82-1.01) | n.s. |
|  | Lifejacket use, Coastguard nearby | | High use (51-100%): 29%; Low use (0-50%): 13%  Risk Ratio: 0.86 (95-%-CI: 0.76-0.98) | p<0.05 |
| Swimming abilities | Lifejacket use, Perceived swimming ability: Beginner/non-swimmer | | High use (51-100%): 17%; Low use (0-50%): 6%  Risk Ratio: reference | n/a |
|  | Lifejacket use, Perceived swimming ability: Intermediate | | High use (51-100%): 50%; Low use (0-50%): 52%  Risk Ratio: 1.24 (95-%-CI: 1.02-1.52) | p<0.05 |
|  | Lifejacket use, Perceived swimming ability: Expert | | High use (51-100%): 33%; Low use (0-50%): 42%  Risk Ratio: 1.25 (95-%-CI: 1.03-1.53) | p<0.05 |
|  | Lifejacket use, 0 times swam in the pool last 12 months | | High use (51-100%): 40%; Low use (0-50%): 41%  Risk Ratio: reference | n/a |
|  | Lifejacket use, 1 - 12 times swam in the pool last 12 months | | High use (51-100%): 46%; Low use (0-50%): 38%  Risk Ratio: 0.91 (95-%-CI: 0.85-0.99) | p<0.05 |
|  | Lifejacket use, 13 or more times swam in the pool last 12 months | | High use (51-100%): 14%; Low use (0-50%): 21%  Risk Ratio: 0.96 (95-%-CI: 0.88-1.04) | n.s. |
|  | Lifejacket use, 0 times swam in open waters last 12 months | | High use (51-100%): 47%; Low use (0-50%): 30%  Risk Ratio: reference | n/a |
|  | Lifejacket use, 1 - 12 times swam in open waters last 12 months | | High use (51-100%): 41%; Low use (0-50%): 42%  Risk Ratio: 1.08 (95-%-CI: 0.99-1.18) | n.s. |
|  | Lifejacket use, 13 or more times swam in open waters last 12 months | | High use (51-100%): 14%; Low use (0-50%): 21%  Risk Ratio: 0.96 (95-%-CI: 0.88-1.04) | n.s. |
| Type of activity | Lifejacket use, Water-skiing, tubing, swimming or diving | | High use (51-100%): 8%; Low use (0-50%): 15%  Risk Ratio: reference | n/a |
|  | Lifejacket use, Fishing, hunting or crabbing | | High use (51-100%): 58%; Low use (0-50%): 52%  Risk Ratio: 0.98 (95-%-CI: 0.85-1.13) | n.s. |
|  | Lifejacket use, Pleasure or cruising | | High use (51-100%): 23%; Low use (0-50%): 26%  Risk Ratio: 0.95 (95-%-CI: 0.86-1.06) | n.s. |
|  | Lifejacket use, Transportation, maintenance, storage, other | | High use (51-100%): 11%; Low use (0-50%): 7%  Risk Ratio: 0.95 (95-%-CI: 0.79-1.14) | n.s. |
| Water/Weather conditions | Lifejacket use, Daily high temperature | | High use (51-100%): 66.7F (SD:11.6); Low use (0-50%): 69.9F (SD:11.5)  Risk Ratio: 1.04 (95-%-CI: 1.01-1.07) | p<0.05 |
|  | Lifejacket use, Choppy water | | High use (51-100%): 65%; Low use (0-50%): 77%  Risk Ratio: 1.16 (95-%-CI: 1.06-1.27) | p<0.05 |
| Waterway type | Lifejacket use, Salt water | | High use (51-100%): 64%; Low use (0-50%): 55%  Risk Ratio: 0.94 (95-%-CI: 0.0.88-1.00) | n.s. |
| **Reference** | ***Quistberg et al (2014b)*** | | | |
| **Factor** | **Outcome** | | | |
| Alcohol and other drugs | After drinking people do ‘stupid things’ and are as a result less likely to stay safe by wearing a lifejacket. | | | |
| Boating abilities | Low levels of experience are perceived to lead to overconfidence and as a result in lower use of lifejackets. | | | |
| Comfort of lifejackets | Lifejackets are generally perceived to be uncomfortable whereas inflatable lifejackets in particular are perceived to be more comfortable despite higher costs. | | | |
| Legislation/Regulation | Mandatory legislations, i.e. mandatory for all or at least for specific age groups, might be helpful to increase the use of lifejackets. | | | |
| **Reference** | ***Redwood et al (2009)*** | | | |
| **Factor** | **Outcome (specific)/Group** | | **Result** | **Sig.** |
| Age | Lifejacket use (always), all age groups | | 51.5% (n=1476) | p<0.05 |
|  | Lifejacket use (always), 18 – 29 years | | 42.0% (n=373) |  |
|  | Lifejacket use (always), 30 – 39 years | | 48.1% (n=290) |  |
|  | Lifejacket use (always), 40 – 59 years | | 58.6% (n=646) |  |
|  | Lifejacket use (always), 60 years and older | | 60.9% (n=167) |  |
| Gender | Lifejacket use (always), Male | | 45.2% (n=576) | p<0.05 |
|  | Lifejacket use (always), Female | | 56.5% (n=900) |  |
| Region | Lifejacket use (always), Southcentral | | 64.0% (n=523) | p<0.05 |
|  | Lifejacket use (always), Southeast | | 51.9% (n=323) |  |
|  | Lifejacket use (always), Southwest | | 44.1% (n=629) |  |
| **Reference** | ***Strayer et al (2010)*** | | | |
| **Factor** | **Outcome (specific)/Group** | | **Result** | **Sig.** |
| Gender | Lifejacket use, Male | | 18% | n.s. |
|  | Lifejacket use, Female | | 21% |  |
| Race/Ethnicity | Lifejacket use, Native Alaskans | | 8% | n.s. |
|  | Lifejacket use, All other | | 23% |  |
| **Reference** | ***Treser et al (1997)*** | | | |
| **Factor** | **Outcome (specific)/Group** | | **Result** | **Sig.^^^** |
| Age | Lifejacket use: All groups | | 1992: 19.9% (n=218); 1994: 31.3% (n=937) | p<0.001 |
|  | Lifejacket use: Adults | | 1992: 14.2% (n=131); 1994: 24.6% (n=628) | p<0.001 |
|  | Lifejacket use: Children | | 1992: 68.0% (n=70); 1994: 68.5% (n=263) | n.s. |
|  | Lifejacket use: Toddlers | | 1992: 58.3% (n=7); 1994: 86.6% (n=46) | p<0.022 |
|  | Lifejacket use: Adult males | | 1992: 13.6% (n=96); 1994: 22.8% (n=360) | p<0.001 |
|  | Lifejacket use: Adult females | | 1992: 10.8% (n=26); 1994: 23.4% (n=206) | p<0.001 |
|  | Lifejacket use: Under 15 years (males) | | 1992: 67.1% (n=47); 1994: 70.2% (n=186) | n.s. |
|  | Lifejacket use: Under 15 years (females) | | 1992: 66.7% (n=30); 1994: 71.1% (n=113) | n.s. |
| Gender | Lifejacket use: Males | | 1992: 18.2% (n=134); 1994: 29.6% (n=548) | p<0.001 |
|  | Lifejacket use: Females | | 1992: 20.4% (n=55); 1994: 30.3% (n=316) | p<0.001 |
| **Reference** | ***Wintemute et al (2013)*** | | | |
| **Factor** | **Outcome (specific)/Group** | | **Result** | **Sig.** |
| Age | Lifejacket use, under 1 year | | 55.6% (n=15)  OR: 7.7 (95-%-CI: 3.3-18.1); AOR: 9.1 (95-%-CI: 3.8-21.7) | p<0.05 |
|  | Lifejacket use, 1 – 4 years | | 37.6% (n=208)  OR: 3.3 (95-%-CI: 1.3-4.8); AOR: 3.5 (95-%-CI: 2.4-5.1) | p<0.05 |
|  | Lifejacket use, 5 – 10 years | | 29.4% (n=252)  OR: 2.3 (95-%-CI: 1.6-3.3); AOR: 2.4 (95-%-CI: 1.7-3.5) | p<0.05 |
|  | Lifejacket use, 10 – 13 years | | 14.6% (n=42)  OR: reference; AOR: reference | p<0.05 |
| Gender | Lifejacket use, Female | | 27.9% (n=230)  OR: 0.8 (95-%-CI: 0.7-1.0); AOR: 0.8 (95-%-CI: 0.6-1.0) | n.s. |
|  | Lifejacket use, Male | | 31.7% (n=278)  OR: reference; AOR: reference | n.s. |
| Race/Ethnicity | Lifejacket use, African-American | | 29.0% (n=64)  OR: 0.8 (95-%-CI: 0.6-1.1); AOR: 0.8 (95-%-CI: 0.6-1.2) | n.s. |
|  | Lifejacket use, Asian | | 19.5% (n=15)  OR: 0.5 (95-%-CI: 0.3-0.9); AOR: 0.5 (95-%-CI: 0.3-0.9) | p<0.05 |
|  | Lifejacket use, Hispanic | | 29.2% (n=238)  OR: 0.8 (95-%-CI: 0.7-1.1); AOR: 0.9 (95-%-CI: 0.7-1.1) | n.s. |
|  | Lifejacket use, White | | 18.2% (n=4)  OR: 0.5 (95-%-CI: 0.2-1.4); AOR: 0.4 (95-%-CI: 0.1-1.0) | n.s. |
|  | Lifejacket use, Uncertain | | % (n=)  OR: reference; AOR: reference | n.s. |

Legends: *Figures in this publication are confusing and unclear. Information in figures is insufficient at times. ^$^Exact numbers not available in publication. ^#^Differences between high use and low use groups. ^^^Difference between years. PWC = Personal Watercraft
